# Mammary Paget disease presenting with nipple erosion and crusting: a rare clinical image

**DOI:** 10.11604/pamj.2026.53.73.50497

**Published:** 2026-02-10

**Authors:** Garima Gupta, Shweta Parwe

**Affiliations:** 1 Department of Panchakarma, Mahatma Gandhi Ayurveda College Hospital and Research Centre, Salod (Hirapur), Datta Meghe Institute of Higher Education and Research, Wardha, Maharashtra, India

**Keywords:** Breast neoplasm, mammary Paget, nipple erosion, nipple lesion, nipple crusting

## Image in medicine

A 55-year-old female presented for dermatologic evaluation of a persistent unilateral nipple lesion. No detailed past medical history was available at the time of assessment. Clinical examination revealed erythematous, eroded, and crusted changes involving the nipple, with a centrally ulcerated area and surrounding induration. The adjacent areola showed hyperpigmentation and coarse surface wrinkling suggestive of chronic involvement. A second image demonstrated a nipple-centred eroded plaque with focal crusting. The diagnostic approach was based on the characteristic nipple-centred erosion, crusting, and chronic architectural distortion, which raised strong suspicion of mammary Paget disease, a rare intraepidermal adenocarcinoma frequently associated with underlying breast carcinoma. The patient was advised to undergo further evaluation, including breast imaging and histopathological confirmation to assess associated malignancy. The patient was referred for multidisciplinary evaluation and oncologic work-up, with planning for definitive management following histopathological confirmation. At short-term follow-up, the lesion persisted without clinical resolution, and further investigations were ongoing.

**Figure 1:**
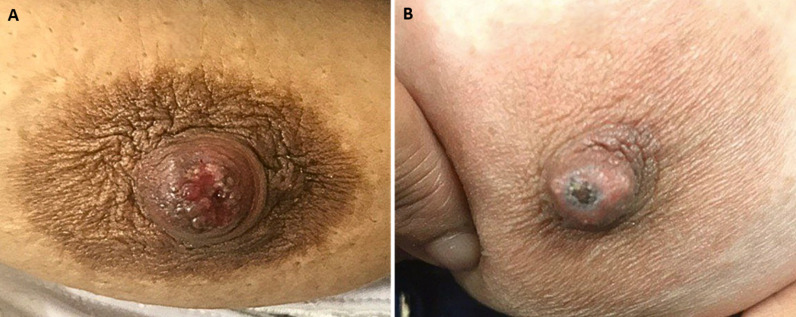
A) erythematous, eroded, and crusted lesion involving the nipple with surrounding areolar changes; B) nipple-centred ulcerated and crusted plaque demonstrating classic morphology of mammary Paget disease

